# Notes and Comments

**Published:** 1922-04

**Authors:** 


THE
### HOSPITAL and HEALTH
REVIEW

### THE ECONOMIC VALUE OF HEALTH SERVICES.

APART from a falling death rate and the alleviation of an incalculable amount of suffering, the experience of the last thirty years proves conclusively that the improvements in sanitary administration and health services have had a definite economic value which is immeasurably greater than their actual cost. The death rate has been definitely decreased by a substantial proportion. This saves an enormous amount of useful and productive labour by preserving to the community the labour of many thousands already skilled in various walks of life. The reduction may fairly be ascribed to the efforts made to deal in a systematic way, during the last half-century, with insanitary conditions throughout the country, first by local boards of health and, later, by municipal bodies.

The matter may be considered under two heads—(*a*) the continuance of life, and (*b*) the reduction of sickness and disablement. An enormous reduction has been made in sickness and disease. Many diseases are now controllable by efficient sanitary administration—*e.g.*, improved public water supply and sewage disposal and protection of milk and other foods—and some diseases—*e.g.*, typhus fever—have almost entirely died out. As a result of efforts to improve the health of the people, mortality from nearly all the principal diseases has declined materially in 30 years. Who will venture to estimate the money value alone of the difference between the sickness and mortality rates in the working population in 1870 and 1920 ? Whatever the gain already achieved, it has been estimated that at least 13,537,388 weeks' work is still lost on an average every year through sickness, or a period of upwards of 260,000 years. That is to say, the nation loses annually the equivalent of the work of 260,000 persons and the labour and expense involved in their care during the 13½ million weeks of their sickness.

Measures can and are being taken to ensure that as far as possible every child shall have at least a "sporting chance." Formerly not only was there a huge infantile mortality rate, but many children before they started life were seriously handicapped by preventible diseases and afflictions. Many were either crippled or blinded and their whole lives made almost useless to the community. The improvement is largely the result of the maternity and child welfare centres and clinics. It would be difficult to estimate in terms of figures the resultant gain to the nation.

Notwithstanding the improvement shown, there is still a vast amount of maternity and child welfare work to be done. We lose by death not less than 60,000 infants a year. Upwards of 3,000 mothers also die every year in the fulfilment of one of their principal and most vital duties to the State. In the next place there is a large volume of abortion miscarriage, still-birth and ophthalmia neonatorum, much of it directly avoidable. Again, a substantial number of mothers are so injured or disabled in pregnancy or childbirth as to make them chronic invalids—an excessive burden of preventable invalidity, incapacitating in its immediate and deterrent in its remote effects. It would be difficult to over-estimate the vast mass of suffering, disability and incapacity which is thus caused and ought not to be caused. This problem can be met only by an effort which must be comprehensive and not confined to one or two methods.

It must be concerned with the efficiency and adequacy (1) of methods for the supervision of pregnancy, (2) of midwifery, (3) of maternity homes and hospitals, (4) of home nursing and health visitation, (5) of infant welfare centres and institutions, and (6) of the sound administration and wise use of maternity benefit.

A national campaign against "the white scourge" was commenced in 1912, following the report of a committee upon the consideration of a general policy in respect of the problem of tuberculosis in the United Kingdom in its preventive, curative and other aspects, which should guide the Government and local bodies in making or aiding provision for the treatment of tuberculosis in sanitoria or other institutions or otherwise. Schemes for the institutional treatment of tuberculosis have now been formulated by practically every county council and county borough council in England, and in the great majority of areas agreements have been made with the insured committees under which the scheme is available for insured persons recommended for sanatorium benefit. The accommodation in sanatoria and hospitals belonging to local authorities and voluntary bodies has been increased from under 5,700 beds in January, 1912, to 8,888 beds in August, 1914, and to 13,523 beds at the end of March, 1919. The result of these measures is shown by the fact that the total number of cases of all forms of tuberculosis has been reduced from 135,041 in 1913 to 82,050 in 1919, or very nearly 40 per cent.

We are in fact sowing wiser than we know, and future generations will be grateful to us for the public health work of the present—one of the most real economies, if wisely administered.

---

### DENTAL FEES.

According to the regulations of the Dental Board of the United Kingdom, it is proposed to charge dentists who go on the Register in future not only a £5 registration fee, but an annual retention fee of £5. On the assumption that 10,000 dentists will register, the Dental Board will obtain a revenue of £50,000 a year. Considerable objection has been raised to this provision, especially by those dentists who, up to the present, have been unregistered.

Any unprejudiced person must consider this objection to be absurd and unreasonable. Surely it is not unfair to charge a man who is going on the register £5 a year, especially if he has not passed any medical examination or taken any degree. The dentist is obtaining a very good bargain from his point of view and is being protected from the competition of the blacksmith with his pincers and other unqualified quacks. In other professions annual fees are paid. The practising solicitor pays £9 a year as a practising fee; the barrister certainly pays no annual fee, but his call fees with stamp duties amount to over £100 a year. This sum alone, to say nothing of the expenses of being called to the Bar, would pay the dentist's annual registration fee for over twenty years.

Those who are objecting to this sensible provision of the Dental Board appear to think that the money will be used by the members of the Board for either their own remuneration or their personal comfort. This is quite inaccurate. It is true, certainly, that the Dental Board ought to be provided with decent headquarters, which are needed not only for the accommodation of the staff, but because in the future the Board will have to conduct judicial proceedings and hold examinations very much on the same lines as those held now by the General Medical Council. There must be for this purpose a hall and conference rooms as well as offices. In the premises at present used there is hopeless congestion. The expenditure on new buildings, however, should not use up more than the income of the first two years.

The fundamental need for annual fees is that there is an overwhelming necessity for acquiring means for the encouragement of dental education, training and research. If a number of unqualified men are to be dumped on the Register, clearly post-graduate instruction must be provided. This problem is as acute in dentistry as it is in medicine. Many of these unqualified dentists have not given three years of their lives to study in order to obtain degrees. Why should they not now contribute, in return for advantages gained, towards the endowment of dental education?

Research into dental diseases is also imperative. Much valuable work has been done recently in this direction, but even now the world is ignorant of the causes that produce dental caries and of the relation between different kinds of diet and healthy teeth. Some of the funds that will result from this fee will no doubt be devoted to this object.

Another reason why the Dental Board should be provided with an income is that scholarships may be established to make it easy for promising but impecunious youths to qualify as dentists. It is a democratic proposal to take money from those who are now being given special privileges by the State in order to widen the area of recruitment for the future.

It is to be hoped that those responsible will stand firm by their proposal to impose this fee. Medical education is already endowed either through the hospitals or by special Government grant. There is no endowment for dental education, and if such a fund were not created the permanent work of the Dental Board would become ineffectual.

---

### THE NARROWING ENGLISH FACE AND ADENOIDS.

Sir Arthur Keith and Mr. G. G. Campion have published in the "Dental Record" an important paper on the growth of the face in which they come to some interesting conclusions. From observations of the former on prehistoric English skulls they are convinced that in a considerable proportion of the modern population there is a tendency for the face to become longer and narrower. This tendency is directly related to narrowing and arching of the palate. Bony matter is apparently laid down to about the same amount in these long, contracted, hatchet faces as in the shorter, wider, more primitive prehistoric faces, but from some alteration in the mechanism of growth new bone which was formerly laid down to add to the width of the face is, in a large proportion of present-day people, deposited so as to increase the length at the expense of the width. With this reduction in width comes a pinching of the facial skeleton from side to side leading to the irregular projection of points situated in the middle line of the face. They are convinced that the so-called "adenoid face," the narrow, deep palatal arch, the irregular eruption of teeth, are not due to a simple mechanical cause such as the presence of enlarged tonsils, or of adenoid vegetations in the respiratory tidal way. We are dealing with an arrest or a disturbance of the elaborate machinery which underlies facial growth. There is evidence showing that this mechanism is influenced by the functional state of the pituitary and of the sexual organs. The authors regard enlargement of the tonsils, the growth of adenoid vegetations in the naso-pharynx, and disordered or irregular growth of the jaws and face as manifestations of a single pathological state. What the exact nature of this underlying pathological condition may be they are not yet in a position to decide, but the close relation between bone-formation and bone-growth and the action of the glands of internal secretion on the one hand, and the frequency with which hypertrophy of the lymphoid system is accompanied by defective formation of the bone on the other renders it not difficult to believe that irregularities of the jaw and the development of adenoids may result from a nutritional disturbance. The explanation may come through the kind of research now being conducted by Colonel McCarrison, who has shown that a diet deficient in vitamines will affect the normal working of the hormone growth systems of the body. Vitamines appear to act directly on the glands of internal secretion. The defects which are so frequent in the growth of the English, or rather Nordic, face may ultimately prove to be the result of some deficiency or error in the dietary on which infants and youths are reared. No doubt, too, modern dietary is of a kind which leaves teeth, jaw and muscles of mastication imperfectly exercised. The physical stimuli necessary for the normal growth of bone are missing. This, too, may be a factor in degeneration of the jaws.

---

### INSURANCE ECONOMIES.

THE Geddes Committee recommended that there should be an increase of one penny in the weekly contribution under the National Health Insurance Scheme to be divided equally between employers and employed. The object of this was to relieve the Exchequer of certain extra charges in connection with medical benefit. When this proposal was submitted to the representatives of approved societies of all types on the Consultative Council of the Ministry of Health, the Council were unanimously of opinion that the present time was inopportune for any increase in the weekly contributions. They therefore submitted to the Government an alternative offer to effect the economies recommended by the Geddes Committee by using the accumulated surpluses of the Societies. This offer has been accepted.

It is important that it should be generally realised that this arrangement, which will continue until December 31, 1923, will not affect the additional benefits that have been approved. There has been a mistaken idea in certain quarters that in consequence of this decision, the disposable surpluses, revealed at the first valuation of the societies, will not be used in providing additional benefits as contemplated. This is not the case.

The new proposal will not affect the 8,000 schemes that have already been approved, and many of which are now in operation. Under these the vast majority of approved societies and branches are providing their members with additional benefits either in the form of increases in the ordinary rates of sickness, disablement and maternity benefits, or in many cases in the form of dental treatment, hospital treatment or nursing services.

In addition to the disposable surpluses which have been applied in the provision of these additional benefits, societies have also reserve surpluses which were carried forward, as well as their accumulated Contingencies Funds, to which recourse was not necessary. It is from these two sources that the cost, which has hitherto fallen on the Exchequer, is to be defrayed until the end of next year, and the total amount available far exceeds the total cost involved.

Both those responsible for the administration of National Health Insurance and of the approved societies are to be congratulated upon this satisfactory decision. It will be necessary, however, that before December 31, 1923, the whole position as to the case of medical benefit and the manner in which it is to be made shall be reviewed.

---

### THE EXCLUSION OF WOMEN STUDENTS FROM THE LONDON HOSPITAL.

THE London Hospital has decided to take no more women medical students. In a press interview the Chairman, Lord Knutsford, states that the reason is not that they have proved unsatisfactory students, but that the difficulties of educating them with men have proved too great. They were first admitted in 1908. It was then found that a number of men students would not join the hospital if they were to be taught with them. The hospital staff have found that there are great difficulties in teaching certain subjects to mixed classes. This decision has aroused a storm of controversy, and contrary experience has been published from many sources. Dr. W. J. Fenton, Dean of Charing Cross Hospital, has never found difficulties. He argues that in medical education and science sex does not enter at all. At Edinburgh University, where there are about 400 women medical students, no difficulty is found in instructing them in the same classes as men. The only class in which they are taken separately is midwifery. The same view is taken in some other metropolitan schools, and in those of Aberdeen, Belfast, Birmingham, Manchester, Leeds, Glasgow, Cardiff and Dublin. But at Dundee and Manchester sympathy has been expressed with the views entertained at the London Hospital. The suggestion, which has been made by some writers, that the decision of the London Hospital is due not to the reason given but to male jealousy of female competition in medicine, appears to be quite unfounded. Replying to it, Lord Knutsford states that the decision of the London Hospital was not instigated by the staff or due to any such jealousy. Nor was the aspect of the question which the Press have made the most of, *i.e.*, the difficulty of teaching mixed classes on certain subjects—the deciding factor. There were many other difficulties in having a mixed school. But the one which influenced the Hospital most was that it was found that the best students much preferred to go to medical schools and hospitals where there were no women. The Hospital could not risk ruining the school for men for the problematical benefit to women students who have accommodation elsewhere. Lord Knutsford is strongly in favour of women being doctors and pays a handsome tribute to their work in consequence of what he saw of Dr. Flora Murray's and Miss Garrett Anderson's War Hospital in Endell Street, where there was no man on the staff. They made history by their pioneer work. Their hospital, in his opinion, was equal to the best, and was the most popular of all.

---

### THE QUARRELS OF THE ANTI-VENEREAL DISEASE SOCIETIES.

IN politics the party system may be useful; its encouragement of reciprocal criticism may be of constructive value. But it is very certain that the campaign against venereal disease in this country has suffered greatly from the mutual hostility of the National Council for Combating Venereal Diseases (N.C.C.V.D.) and the Society for Prevention of Venereal Disease (S.P.V.D.). Recently in the "Lancet" representatives of both societies have claimed some sort of kinship with the older German society, *Deutsche Gesellschaft zur Bekämpfung der Geschlechtskrankheiten* (D.G.B.G.). It is eloquent of the change which has taken place since the war in the attitude of this country towards Germany that such claims should be made. For the matter in dispute is one of morals and ethics, and this country has not always accepted the German code of morals as the last word on the subject. Hitherto the D.G.B.G. has been able to present a fairly united front, and there is no doubt that it has done excellent work, although the members of its executive have not always seen eye to eye in matters of policy, and it has had to walk warily so as not to offend the susceptibilities of moral welfare and evangelical societies in Germany. Its unity is threatened by the present clamour of the English societies, whose quotations, from the utterances of various officials within the D.G.B.G., are calculated to split this society by emphasising the differences of opinion within its ranks. The point at issue is, of course, over the ethics of personal prophylaxis: can adolescents be taught how to use devices for preventing infection and be freely supplied with such devices without injury to their morals? Elementary humanity insists on the relief of suffering and the cure of disease already contracted. But there are many who boggle at the idea of a boy's equipment, as he goes out into the world, including a tube of calomel ointment with minute and tendencious instructions as to its use.

---

### THE ROCKEFELLER GIFT.

ANOTHER link of friendship has been formed across the Atlantic by the generous offer of the Trustees of the Rockefeller Foundation to provide two million dollars towards the cost of building and equipping an Imperial Institute of Hygiene in London. As officially announced, the British Government has accepted the offer, on the understanding that it will provide for the staffing and maintenance of the School.

This Institute will become a centre from which benefits will extend to all parts of the world, for there instruction will be given in public health, forensic medicine and industrial medicine, and in medical ethics and economics. It is expected that the students will include qualified men living in this country or in any part of the British Empire, foreign graduates living for a time in London, officers of our Army, Navy and Air Force, and any members of the Overseas medical services who are on study leave.

Two men on this side deserve the thanks of their fellow-countrymen for the work that they have done in making such an Institute possible. The Earl of Athlone, who presided over the Post-Graduate Medical Committee last year, is partly responsible for the original proposal being publicly adopted. It is well known, too, that Sir George Newman, the Chief Medical Officer of the Ministry of Health, whose wide vision is helping forward the making of a healthier nation, had a large share in the scheme. Sir Alfred Mond had taken up the proposal with a committee of experts, but progress was stopped owing to financial difficulties. These the generosity of the Rockefeller Foundation has overcome.

It is now almost a certainty that the Institute will be erected in London and will be controlled by a general governing body. It will probably occupy to the University of London a position analogous to that held by the Institute of Historical Research. It is expected, however, that some two years will elapse before the proposal will materialise, for well-equipped laboratories have to be built and expert staffs selected.

### CLEANER MILK.

IN our next issue will be found an article by Mr. Wilfred Buckley, whose devoted work for the National Clean Milk Society has so much assisted in the education of public opinion. Those who believe with him that clean milk is vital for health await anxiously the production of the promised legislation on the subject. It can be taken for granted that the 1915 Act as it stands cannot be brought into operation because of the cost. There must be suspension of some or all of the provisions of that Act. At the same time it would be a disaster if the experiments in grading milk came to an end next August. Although clearly in the present financial situation any legislation that involves the appointment of new officials or of fresh expenditure must be postponed, it is to be hoped that Sir Alfred Mond will recognise that public opinion is with him in demanding that more safeguards be provided for the cleanliness of the milk supply. It is expensive to supervise all the processes of the distribution of milk, but if existing powers be utilised to the full the product as sold might be much improved in quality.

### SAVING WATER.

ALL the experts are of opinion that during the coming year there is bound to be a serious shortage of water. In 123 districts the authorities responsible have reported that the supply available is far below the normal. In view of the importance of clean water for the preservation of health, now is the time, especially in rural districts, for provision to be made for the utilisation of rain-water for garden purposes and for dome tic use. With a roof area of 100 square feet and a tank capacity of 67 cubic feet, the daily yield from rain in a district with an annual rainfall of 25 inches is equivalent to 2·8 gallons. A galvanised iron tank for the storage of rain-water to hold 100 gallons can be bought at 25s., and it may be wise for those responsible for health in the country districts where serious shortage is expected, to conside: the storage of rain-water in order to conserve deep water supplies for drinking purposes. In view of the probability that the water situation will be serious during the summer, arrangements have been made for articles on the subject to be published next month in this Review. Sir Alexander Houston, Director of Water Examination to the Metropolitan Water Board, will write on some of the practical aspects of purifying water. No man in this country can write with more experience of the problem. He was one of the experts at Cairo in 1907, was at Ottawa in 1913, and has been on almost every expert committee dealing with water since he was bacteriologist to the Royal Commission on Sewage Disposal. Another contributor, also an expert with a world-wide reputation, well known to all the water authorities in this country, has promised to write a special article that should prove of great assistance to those districts that are faced with a water shortage in the coming months.

### THE MOTHER OF MEDICINE.

SIR GEORGE NEWMAN, Emeritus Lecturer on Public Health at St. Bartholomew's Hospital, has published a monograph, "A Century of Medicine at Padua," published by the British Periodicals, Ltd., 170 Fleet Street, price 1s. This year is the 700th anniversary of the founding of Padua in 1222. In 1595, when Shakespeare wrote of "Fair Padua, nursery of arts," the first Medical School of Italy at Padua was at its zenith. Thence the revival of medicine passed to Leyden, thence to Edinburgh, and so to the great medical schools of the New World—to Philadelphia, Columbia and Harvard. Sir George Newman always writes in a graceful style, which adorns a wealth of wisdom and expresses a vision that will make for him a place in medical history. This study is both an excursion into literature and a sagacious contribution to the library of public health.

### THE LATE SIR HARCOURT CLARE.

THE municipal world is the poorer by the recent death of Sir Harcourt Clare, the well-known Clerk to the Lancashire County Council. He was recognised as one of the greatest authorities on local government law. He took an important part in the drafting and bringing into operation of the Education Act of 1902. As Clerk to the Lancashire Asylums Board his experience of the administration of our mental hospitals was authoritative. He began his municipal career as Assistant Town Clerk of Liverpool in 1880; he became Town Clerk in 1885, and in 1889 was unanimously appointed Clerk of the Lancashire County Council. He was universally respected, and his death is a serious loss to public health administration, as well as to local government.

### LUMP SUMS FOR HEALTH.

THE recommendation of the Geddes Committee that in future the national Exchequer shall give financial assistance to the public health services in the form of a lump sum rather than on a percentage basis is bound to result in considerable modifications in local administration. There is ample room for economy in these matters and for the abolition of some of the absurdities which can be seen, for example, in badly organised infant welfare centres. The proposed system, which is simply a reintroduction of the method which prevailed before 1914, should also encourage some business men to be prepared to give their time upon Health Committees throughout the country. Recently many Councillors and others on such Committees have complained that they were becoming merely local instruments of Whitehall officials, who left them little local responsibility and imposed large numbers of checks and returns.

Under the system proposed by the Geddes Report each Health organisation belonging to a local authority, if the Government accepts the recommendation, will know exactly what sum is available for the year and will then have to ensure that each pound expended produces its full value.

### INDUSTRIAL NURSING.

INDUSTRIAL nursing was touched on by Sir Alfred Herbert, chairman of the Coventry and Warwickshire Hospital, in his speech at the recent annual meeting. He said that employers could help hospitals not only by giving subscriptions and encouraging them among their employees, but by appointing a nurse in every industrial concern to render first aid and deal with accidents. Where nurses had been engaged in many businesses of which he had knowledge, he was astonished at the valuable amount of work that they did, and the large call made upon their services. Many cases which must otherwise go to hospital and occupy valuable time could be dealt with by a nurse, and thereby save the hospital both time and expense. In his experience of the city, the number of accidents had decreased. The machinery was protected better and run with more intelligence. But the crown of this improvement would be the appointment of a nurse to deal with slight accidents in all factories. Sir Alfred's suggestion is being followed, and the example of America is penetrating here. For many nurses, too, such work is congenial, if only because it is centralised and avoids the going out in all weathers, which often makes district nursing so hard.

### THE VOLUNTARY BOARDER.

IN an unusually interesting report on the work of the Royal Edinburgh Asylum, the Physician-Superintendent, Professor George M. Robertson, discusses the voluntary boarder, the use of psychoanalysis in mental treatment and other matters, with a keenness rarely expressed in these documents. He records that, for the second year in succession, half the patients admitted to Craig House were voluntary boarders. Voluntary admissions to some other institutions were equally high, which showed a widespread change in public feeling. In Scotland this was partly due to the 1913 Act, whereby a written application to a medical superintendent sufficed to gain admission. This meant that half the patients, in the early stages, had some insight into their condition, and allowed themselves to be persuaded by their doctors and relatives to submit to treatment. All this proved treatment without certification to be feasible, and that wholesale certification, before treatment, was harsh and unnecessary. Voluntary admissions encouraged early treatment; certification could be granted only at a late stage. Again, nothing was so conducive to the efficient administration of a mental hospital as the presence of large numbers of voluntary boarders. Their views and criticisms could not possibly be ignored.

But why, Dr. Robertson asks, should these patients be limited to the well-to-do? The poor are debarred at present because the Government give a grant-in-aid to parish councils for the maintenance of certified lunatics, but nothing for the upkeep of voluntary patients. Besides, every certificate recognised by law was a certificate for a definite object: there was no "certificate of insanity." How could a medical man conscientiously certify that a voluntary patient was "a proper person to be detained"? The simple test should be whether a patient conscious of mental symptoms desired treatment and was also alive to the nature and consequence of an application for it.

Dr. Robertson also records the proposal to choose early cases of dementia præcox and to treat them with vaccines, an experiment for which there is every facility at the Royal Hospital, where several cases are already under treatment. Dr. Connell, one of the assistant physicians, has also treated cases by psychoanalysis, and in mild cases obtained favourable results.

### MENTAL HOSPITAL ADMINISTRATION.

THE Committee on the Administration of Public Mental Hospitals is carrying on its work quickly and efficiently. It is to be hoped that, as well as officials, chaplains and nurses who have served in mental hospitals will avail themselves of the Committee's invitation to give evidence. But the Committee has already served a useful purpose in revealing to the public the qualifications of Dr. Lomax in acting as a critic of asylums. His sensational charges have given endless grief and suffering to thousands of relatives. Experience as a temporary assistant medical officer for eighteen months in one asylum and for two months in another asylum under war conditions certainly does not entitle any man to pose as an expert. There are many defects in the present administration of our mental hospitals, but "stunt" methods should not be applied to intricate questions of mental disease.

### THE CLASSIFICATION OF MENTAL CASES.

DR. D. K. HENDERSON, Physician-Superintendent of the Glasgow Royal Asylum, has put in a plea for the better classification of mental cases in the different asylums. He points out the advantages of a systematic grouping of mental disorders, and urges a uniform system of classification. Were one in existence, comparisons between one part of the country and another would be much less tentative and unreliable than is at present the case. In his own institution Dr. Henderson is attempting something of the kind, and since no system can be immediately recommended without discussion and reasoned argument we should welcome information of the system which he is adopting and of the reasons which have led him to prefer it. At present, perhaps, more attention is given in reports to the symptoms of mental disorders than to the careful grouping of the diseases. How far, even with the modern analytic aids to diagnosis, a classification such as Dr. Henderson desires is practical may be a matter for debate.

### HOSPITAL MANNERS.

IS it an unreasonable and unattainable ideal that char-ladies from Lisson Grove should receive the same courtesy and attention in the out-patient department of a general hospital that other ladies of Belgravia enjoy in the consulting-rooms of Harley Street ? To the scoffing snob the answer is no doubt in the affirmative. But there seem to be men in this country who not only preach, but also practise this ideal, and we have this on the authority of Dr. Carl With, a Danish specialist who has recently made a tour of inspection of the principal London hospitals. Writing in " Hospitalstidende," the leading Danish medical journal, he indulges in the candour of the traveller who has returned home and is addressing his own countrymen. Of St. Mary's Hospital he writes :—" The Head of the Dermatological Department is the well-known Graham Little, a God-inspired clinician of the old school. He did everything himself, and the imposing celerity with which he got through his work was marked by a humaneness which was outstanding even for such a country as England. Here it was possible to learn that superciliousness and impatience are unnecessary and, as a rule, betray a fault in the physician rather than in his clientèle. He stated that he regarded it as his chief duty to conduct his hospital practice on the same principles as his private practice—an ideal which many of us would do well to bear in mind." If every employee of a general hospital would remember that duchesses and foreign doctors may, like angels, be entertained unawares, there would be no risk of lapses from the high standard set by Dr. Graham Little.

### VOTES FOR HOSPITALS.

AT the time of the recent London County Council election, a hospital committee-man remarked that, though his institution paid rates at the average sum of £6 per bed, the hospital had no vote. It was a timely reminder, because hospitals have always tried to be assessed at a much smaller sum than at present, and for the current schemes of weekly contributions, their advocates have begun to urge the organisation of districts on the lines of the city wards, sometimes with the aid of municipal or municipal electioneering machinery. Had hospitals votes, which might seem to involve them in the unhappy climate of municipal politics, they could, as large ratepayers, at least press their claims to relief in a manner at present impossible. We doubt, however, if the existing authorities would welcome the vote of corporate bodies like hospitals or business companies.

### HOSPITAL OFFICIALS FOR LOCAL COMMITTEES.

WE notice that one of the new Local Committees appointed under the Voluntary Hospital Commission has chosen a hospital secretary to be its secretary. That his abilities are considerable seems to us a doubtful reason for this appointment. Since the Report of Lord Cave's Committee emphasised the importance of the Local Committees being largely composed of non-hospital men, that co-ordination might be considered impartially, the wisdom of making the Local Committee's secretary a man who is a hospital secretary also remains to be proved. It may work well, but the gentleman's preliminary remarks expressed a doubt that any new money could be raised, which, after all, should not be the attitude of mind expected from the new Committees, one of whose duties will be to foster new sources of income. It seems to us doubtful how far a hospital secretary can take the large view which the Local Committees are intended to foster, and we shall watch with interest how the new appointment works.

### HOSPITAL CO-ORDINATION IN LONDON.

A GOOD instance of the need for co-ordination of hospitals and areas in London is provided by the alleged overlapping between the Royal Northern Hospital and the Prince of Wales's General Hospital, Tottenham. The admitted shortage of hospital beds is nowhere more apparent than in North London, which has grown so enormously of recent years. Of the two hospitals which help to serve this area, the Royal Northern is the greater. Cases consequently tend to flow there, though the Prince of Wales's Hospital is also fully occupied. In these days, however, appeals are made to all areas from which hospital patients are drawn, and it is commonly asserted that the Royal Northern and the Prince of Wales's Hospitals press their claims upon the same districts, perhaps to the disadvantage of the latter. Hitherto a plea for mutual arrangement has been all that could be hoped for, but now that King Edward's Hospital Fund is the Local Committee for London, we hope that the matter will be formally determined. Any early examples of this co-ordination will be watched eagerly ; and the present problem offers an immediate test.

### THE BRISTOL AMALGAMATION.

ONCE again the familiar proposal to amalgamate the Royal Infirmary and the General Hospital at Bristol has been put forward. Though all previous attempts have failed, Mr. Ernest W. Hey Groves recently addressed the British Medical Committee in its favour. His remarks are printed in " The Stethoscope." He argues that the medical school provided the original link which every year of the Bristol University's existence strengthened. A generation ago the rivalry of the two hospitals was pronounced, and co-operation was as little thought of as between the old Whigs and Tories. But during the war a large military hospital was staffed by the medical men of both hospitals, an experience which showed that " the voluntary hospitals were greatly over-staffed," and that combined action was " both easy and beneficial." Medical men and the governors of both hospitals had advised amalgamation. Its postponement was due to "inertia, distrust of co-operation, preoccupation with immediate problems of finance, and practical difficulties." But, Dr. Hey Groves adds, though these facts explain, they do not justify, delay. Amalgamation would improve administration, diagnosis and treatment and clinical teaching. In the meantime several steps towards amalgamation could be usefully taken. It seems to us that those in favour of the scheme, which everywhere is beset with difficulties, should lay their proposals before the new Local Committee appointed by the Voluntary Hospital's Commission. This body exists to encourage co-ordination and would be bound to consider favourably any practical proposals.

### SMALL-POX HOSPITALS AMONG HOUSES.

SHOULD Port Sanitary Hospitals admit cases of small-pox, even if they have been sea-borne? The matter has been discussed in connection with the Port Sanitary Hospital at New Ferry, because that institution is not many hundred yards removed from many of the houses, and it is feared that the reception of such cases may make the hospital a contributory cause of small-pox in the neighbourhood. The County Medical Officer of Health, Dr. Meredith Young, favours the removal of the hospital, since a joint small-pox hospital is not yet established in the neighbourhood. But, whatever may be the evidence in this area, is it yet proved that a small-pox hospital is a source of infection, and so a public danger, unless it is in an area distant from surrounding dwellings?

### HOSPITALS AND UNEMPLOYMENT.

THE pressing question of the unemployed affects hospitals and infirmaries equally though in different respects. Its reduction of hospital income from the weekly contributions of workers in factories is apparent, though less, on the whole, than might have been feared. To the guardians, in regard to their infirmaries, the problem appears in a different form. Thus the West Derby guardians have decided to open tenders for the extension of their nurses' home at Walton rather than wait three months for the anticipated fall in builders' prices. The argument is that the gain in lower tenders three months hence would be swallowed by increased payments to the unemployed in the interval. There is a scarcity of plasterers at present, but these men will not be required, of course, till the work is nearly complete.

### ST. BARTHOLOMEW'S HOSPITAL.

NEXT year the 800th anniversary of the foundation of St. Bartholomew's Hospital will be celebrated. To mark the occasion a general purposes and executive committee has been formed, with the Lord Mayor as permanent chairman of the Grand Committee. The Minister of Health, Sir Alfred Mond, has promised every assistance from his department. The proposals which the executive committee will have to carry out are to prepare an up-to-date account of the Medical College; to hold an historical exhibition, religious services, receptions, dinners and entertainments, and possibly a pageant. A fair and a scientific exhibition are also contemplated. The suggestion for a pageant is particularly interesting because of the movement in favour of a Hospital Pageant for the whole of London, which we have already recorded. The two plans cannot clash, since the Hospital Pageant was proposed for the coming summer and the centenary celebrations of St. Bartholomew's will be held next spring.

### WOULD-BE SUICIDES AS PATIENTS.

CASES of attempted suicide are always an anxiety to hospitals, but few institutions have taken so drastic a step as that lately reported at the Ashton-under-Lyne Infirmary. Within a single week two patients who had attempted to cut their throats were refused admission and had to be taken to the infirmary. Both are stated to have died. The medical staff apparently argue that such cases are ineligible or should be treated elsewhere because of the trouble that they cause to forestall any renewed attempt at self-injury. The committee has informed the police that since the former practice of having a constable in the ward to watch the case has been stopped, the infirmary cannot meet the extra cost involved.

### PAYING PATIENTS IN INFIRMARIES.

SOME interesting returns of the number of vacant beds in the London Poor Law Infirmaries on January 1, 1922, were given at the recent meeting of the London Guardians' Association. When these were compared with the corresponding figures for July 1, 1921, it was resolved "that it would appear from such returns that there is no permanent surplus accommodation . . . other than that required for Poor Law patients." How far the position in London is repeated in the provinces it would be hard to say offhand, but the fact remains that several infirmaries have found it possible to relieve the hospitals' waiting lists. It is well, however, that the limitation on this form of co-operation should be known, since extravagant hopes have sometimes been based upon it.

### THE STANDARD OF CONSIDERATION.

THE Southwark Guardians recently entered a strong protest against the General Lying-In Hospital, York Road, for transferring patients in a critical condition from the hospital to the Guardians' Infirmary at Newington. Of the two cases alleged one was a married girl who was confined two hours after her removal to the Infirmary, and died the next day. The other was a pneumonia patient who was removed late at night. Two of the guardians are life governors of the hospital, and pledged themselves to obtain an explanation. Without prejudging that we may say that, if the standard of consideration is to be kept high in the voluntary hospitals, keen critics are of value if their evidence is not frivolous.

